# Association between stress fracture incidence and predicted body fat in United States Army Basic Combat Training recruits

**DOI:** 10.1186/s12891-018-2061-3

**Published:** 2018-05-22

**Authors:** Joseph J. Knapik, Marilyn A. Sharp, Scott J. Montain

**Affiliations:** US Army Research Institute of Environmental Medicine, 10 General Greene Ave, Natick, MA 01760 USA

**Keywords:** Stress fracture, Military personnel, Body fat, Age, Gender, Body mass index, Height, Weight, Race/ethnicity

## Abstract

**Background:**

A stress fracture (SF) is a highly debilitating injury commonly experienced in United States Army Basic Combat Training (BCT). Body fat (BF) may be associated with this injury but previous investigations (in athletes) have largely used SF self-reports and lacked sufficient statistical power. This investigation developed an equation to estimate %BF and used that equation to examine the relationship between %BF and SF risk in BCT recruits.

**Methods:**

Data for the %BF predictive equation involved 349 recruits with BF obtained from dual-energy X-ray absorptiometry. %BF was estimated using body mass index (BMI, weight/height^2^), age (yr), and sex in the entire population of BCT recruits over an 11-year period (*n* = 583,651). Medical information was obtained on these recruits to determine SF occurrence. Recruits were separated into deciles of estimated %BF and the risk of SFs determined in each decile.

**Results:**

The equation was %BF = − 7.53 + 1.43 ● BMI + 0.13 ● age − 14.73 ● sex, with sex either 1 for men or 0 for women (*r* = 0.88, standard error of estimate = 4.2%BF). Among the men, SF risk increased at the higher and lower %BF deciles: compared to men in the mean %BF decile, the risk of a SF for men in the first (lowest %BF) and tenth (highest %BF) decile were 1.27 (95%confidence interval (95%CI) = 1.17–1.40) and 1.15 (95%CI = 1.05–1.26) times higher, respectively. Among women, SF risk was only elevated in the first %BF decile with risk 1.20 (95%CI = 1.09–1.32) times higher compared to the mean %BF decile.

**Conclusions:**

Low %BF was associated with higher SF risk in BCT; higher %BF was associated with higher SF risk among men but not women.

## Background

A stress fracture (SF) is a serious and debilitating injury commonly seen in United States (US) Army Basic Combat Training (BCT). Previous studies found medically diagnosed SF incidence in BCT to range from 0.8 to 5.1% for men and 1.1 to 18.0% among women [1–7]. SFs often require removal from BCT and supervised rehabilitation that lasts an average of 62 days [8]. Well researched factors increasing susceptibility to SFs in BCT include female gender [2, 3, 9, 10, 11], older age [9, 12, 13], and race/ethnicity other than black [2, 9, 12–15]. The association between SF risk and physical characteristics like height, weight, and body mass index (BMI) is not clear because results were mixed in the various investigations [9, 14, 16–21]. No study to date has examined the association between SF risk and body fat (BF) in BCT, but there have been several investigations examining this association in athletes [22–25]. However, in most of these investigations [22–24] SFs were self-reported and all [22–25] involved few athletes with SFs (*n* ≤ 31) suggesting low statistical power.

BMI is generally taken as a surrogate for BF since it has a close relationship to %BF with correlations on the order of 0.7 [26–28]. However, a particular absolute BMI level does not reflect the same level of body fat in men and women. Examination of the data in a previous BCT study showed that for the same BMI, women had 13 to 15% more BF [26]. Multiple regression equations that have included age, sex, and/or race/ethnicity in addition to BMI have shown improved ability to predict %BF with correlation ranging from 0.81 to 0.91 [28–33].

This investigation examines the ability of BMI, age, sex, and race to predict %BF in a cohort of recruits in BCT. After developing and validating a %BF predictive equation, this investigation uses that equation to examine the association between %BF and SF risk in the entire population of US Army recruits attending BCT over an 11-year period. A specific %BF equation developed using military recruits would appear to be the most appropriate to examine this relationship in BCT. Based on previous studies [28–34] it was hypothesized that an equation using BMI, age, sex, and race/ethnicity should produce a correlation with a direct measure of %BF in the range of 0.81 to 0.91. It was also hypothesized that both high and low %BF would increase the risk of SFs based on studies that have observed this relationship between BMI and %BF [9, 35].

## Methods

This study involved a secondary analysis of data from past investigations that was approved by Human Subjects Protection Office of the US Army Research Institute of Environmental Medicine. Four legacy databases were used, one to develop a %BF prediction equation and the others to apply this predictive equation to examine the relationship between %BF and SF risk. The database used to develop the %BF predictive equation contained date of birth, height, weight, sex, race/ethnicity, and percent body fat (%BF) on 180 male and 169 female BCT recruits. [36] Height was measured with a stadiometer (Model GPM, Seritex Inc., Carlstadt, NJ) and weight with a digital scale (Model 770, Seca Corp., Columbia MD) with subjects in T-shirts, shorts, underclothing, and socks. Date of birth, sex, and race/ethnicity were self-reported. Body fat (BF) was measured with dual-energy X-ray absorptiometry (DXA, Model DPXL, Lunar Corp., Madison WI) [36]. Scanning with the DXA began at the head and progressed in 1-cm segments to the feet with the machine set to the faster 10-min scanning speed. The Lunar software (version 3.6) was used to provide %BF for each participant. Coefficients of variation for measuring fat mass using DXA have ranged from 0.8 to 5.0% [37].

A database of all recruits attending BCT between 1997 and 2007 was compiled from three databases by the Armed Forces Health Surveillance Branch (AFHSB) of the Defense Health Agency. The first of these databases was the Defense Manpower Data Center (DMDC) Master Personnel File which was searched from January 1997 to December 2007 (11-year period) to identify individuals based on their first demographic record (indicating entry into the Army), with a rank of private (E1) to specialist (E4), and 17 to 35 years of age. DMDC database also provided birth year and race/ethnicity. The second AFHSB database was the Defense Medical Surveillance System which provided the injury data on recruits identified in the DMDC database. Specific International Classification of Diseases, Version 9, Clinical Modification (ICD-9) codes were searched for the inclusive time from each recruit’s first DMDC record to 10 weeks after the first DMDC record. This covered the period of BCT plus time in the reception station (where recruits are initially in-processed). SF cases were recruits with the occurrence of an ICD-9 code for pathological fractures or SFs (ICD-9 codes 733.1–733.19 and 733.93–733.98). Pathological fracture codes were included because, prior to 2001, there was no ICD-9 code specifically for SFs and the 733.1–733.19 series contained the codes that clinicians in military facilities were instructed to use for this purpose. It was previously shown that use of the pathological fracture codes rapidly declined shortly after the SF codes were introduced, supporting the concept that clinicians were using the pathological fracture codes for SFs prior to having specific SF codes [38]. The third AFHSB database was that of the Military Entrance Processing Station which provided sex, height, and weight of the recruits. The AFHSC provided the investigators with a single de-identified database linking information from these three databases.

In all databases where available, age was calculated as year of entry into BCT minus birth year. BMI was calculated as weight/height^2^. In the database used to develop the %BF predictive equation, non-linear regression techniques were applied, but they accounted for little additional variance in %BF compared to linear techniques so the latter was used to develop the equation. Predictive residual sums of squares (PRESS) statistics were used to cross-validate the %BF predictive equation [39]. PRESS statistics compute residual errors based on successive removal and subsequent replacement of cases. These PRESS-residuals are summed and replace the residual sum of squares for the entire model to provide a modified correlation coefficient (r-value) and standard error of estimate (SEE).

SF incidence was calculated as: recruits with one or more SFs / total number of recruits X 1000 (cases/1000 recruits). Univariate logistic regression was performed to quantify risk of SFs at deciles of estimated %BF. Simple contrasts were used, comparing the risk at a baseline (referent) decile of %BF (defined with a risk ratio of 1.00) to other deciles of that variable. The baseline decile of %BF was selected such that it included the mean BF value. A multivariate logistic regression model was constructed with SF incidence as the dependent variable and race/ethnicity and %BF as independent variables to see if race/ethnicity modified the relationship between %BF and SF incidence. In both regression models, odds ratios (OR) and 95% confidence intervals (95%CI) were calculated comparing each decile to the referent decile. Statistical Software for the Social Sciences (SPSS, version 24.0) was used to analyze all data.

## Results

The addition of BMI, age, and sex to the regression model accounted for significant portions of the variance in DXA %BF (*p* < 0.05) but race/ethnicity did not (*p* = 0.94). The racial distribution of the BCD sample was 55.01% White, 28.65% Black, 12.32% Hispanic, 2.29% Asian, 1.43% American Indian, and 1 recruit without race specified. The resulting multiple linear regression equation was:

$$ \%\mathrm{BF}=\hbox{-} 7.53+1.43\bullet \mathrm{BMI}+0.13\bullet \mathrm{age}\hbox{-} 14.73\bullet \mathrm{sex}, $$

where BMI was in kg/m^2^, age in years, and sex coded as either 1 (men) or 0 (women). For this equation, the *r* = 0.88 and the SEE = 4.2 %BF. The PRESS estimated r-value was 0.84 and the SEE was 4.2 %BF. Adding interaction terms (age X BMI, age X sex, BMI X sex) accounted for little additional variance (*r* < 0.01). Age and physical characteristics of the men and women in the database used to develop the predictive equation are shown in Table 1.

**Table 1:** Physical Characteristics of Men and Women in Databases

Database	Variable	Men (Mean ± SD)	Women (Mean ± SD)
Body Composition	Age (yr)	22 ± 4	21 ± 3
	Height (cm)	176.5 ± 7.1	163.1 ± 6.2
	Weight (kg)	78.9 ± 12.7	62.6 ± 9.8
	BMI (kg/m2)	25.3 ± 3.7	23.5 ± 3.0
	Body Fat (%)	16.7 ± 6.3	28.8 ± 6.5
Recruit Population 1997–2007	Age (yr)	21 ± 3	21 ± 4
	Height (cm)	175.6 ± 6.9	162.7 ± 6.4
	Weight (kg)	76.1 ± 13.3	61.8 ± 9.5
	BMI (kg/m2)	24.6 ± 3.8	23.3 ± 3.0
	Body Fat (%)	15.7 ± 5.6	28.5 ± 4.3

The database containing all individuals in BCT between 1997 and 2007 included 614,606 recruits. Of these, 30,955 recruits (5.0%) were missing either birth year, gender, height, and/or weight and were not considered further in the analysis. The final dataset used for analysis contained 583,651 recruits, with 475,745 men and 107,906 women. Age and physical characteristics of these recruits are shown in Table 1.

The overall SF incidence was 1.9% for men and 8.0% for women as reported previously [38]. Table 2 shows the SF risk for men and women by deciles of estimated %BF. The men demonstrated a bimodal relationship with elevated SF risk in the lowest and highest %BF groups compared to the decile containing the mean %BF. The women had high SF risk at the lowest %BF decile compared to the mean %BF decile, but the SF risk at the higher BF deciles were similar to the mean decile.

**Table 2:** Association between Stress Fracture Incidence and Body Fat

Sex	Percentile	Body Fat Range (%)	n	Injury Incidence (cases/1000)	Logistic Regression Odds Ratio (95%CI)	*p*-value
Men	1–10	< 8.90	47,545	23.7	1.27 (1.17–1.40)	< 0.01
	11–20	8.90–10.62	47,603	18.2	0.98 (0.89–1.07)	0.62
	21–30	10.63–12.08	47,382	16.6	0.87 (0.79–0.96)	0.02
	31–40	12.09–13.51	47,706	16.3	0.90 (0.82–0.99)	< 0.01
	41–50	13.52–15.05	47,431	16.8	0.89 (0.81–0.98)	0.03
	51–60	15.06–16.73	47,778	18.6	1.00	Referent
	61–70	16.74–18.60	47,579	20.0	1.07 (0.98–1.18)	0.13
	71–80	18.61–20.61	47,525	20.4	1.10 (1.00–1.20)	0.05
	81–90	20.62–23.52	47,605	21.1	1.14 (1.04–1.24)	< 0.01
	91–100	≥ 23.53	47,591	21.4	1.15 (1.05–1.26)	< 0.01
Women	1–10	< 22.90	10,779	90.1	1.20 (1.09–1.32)	< 0.01
	11–20	22.90–24.56	10,802	81.4	1.07 (0.97–1.18)	0.18
	21–30	24.57–25.97	10,805	80.2	1.05 (0.95–1.16)	0.32
	31–40	25.98–27.23	10,752	79.6	1.04 (0.95–1.16)	0.38
	41–50	27.24–28.53	10,712	76.6	1.00	Referent
	51–60	28.54–29.55	10,827	81.0	1.06 (0.96–1.18)	0.22
	61–70	29.56–30.78	10,842	80.8	1.06 (0.96–1.17)	0.24
	71–80	30.79–32.21	10,788	72.8	0.95 (0.86–1.05)	0.30
	81–90	32.22–34.20	10,793	77.3	1.01 (0.92–1.12)	0.83
	91–100	≥ 34.21	10,806	79.2	1.04 (0.94–1.15)	0.45

Table 3 shows that the addition of race to the logistic regression model did little to modify the relationship between %BF and SF risk. Race was an independent risk factor in the multivariate model with black recruits having lower SF risk than other race/ethnic groups.

**Table 3:** Multivariate Association between Stress Fractures, Body Fat, and Race/Ethnicity

Sex	Variable	Strata	n	Injury Incidence (cases/ 1000)	Multivariate Logistic Regression Odds Ratio (95%CI)	*p*-value
Men	Body Fat	< 8.90%	47,545	23.7	1.27 (1.16–1.39)	< 0.01
		8.90–10.62%	46,603	18.2	0.98 (0.89–1.07)	0.61
		10.63–12.08%	47,382	16.6	0.89 (0.81–0.98)	0.02
		12.09–13.51%	47,706	16.5	0.89 (0.80–0.97)	0.01
		13.52–15.05%	47,431	16.6	0.89 (0.81–0.99)	0.04
		15.06–16.73%	47,778	18.7	1.00	Referent
		16.74–18.60%	47,579	20.0	1.07 (0.97–1.17)	0.17
		18.61–20.61%	47,525	20.4	1.08 (0.99–1.19)	0.09
		20.62–23.52%	47,605	21.1	1.13 (1.03–1.23)	0.01
		≥ 23.53%	47,591	21.4	1.17 (1.07–1.28)	< 0.01
	Race/Ethnicity	Black	72,155	12.2	1.00	Referent
		White	324,089	21.0	1.72 (1.60–1.85)	< 0.01
		Hispanic	52,684	19.3	1.56 (1.43–1.71)	< 0.01
		Asian	15,439	17.0	1.38 (1.20–1.59)	< 0.01
		Am Indian	4812	21.8	1.75 (1.42–2.15)	< 0.01
		Other	1393	25.8	2.12 (1.52–2.98)	< 0.01
		Unknown	5173	16.0	1.31 (1.04–1.64)	0.02
Women	Body Fat	< 22.90%	10,779	90.1	1.22 (1.11–1.35)	< 0.01
		22.90–24.56%	10,802	81.4	1.09 (0.98–1.20)	0.10
		24.57–25.97%	10,805	80.2	1.06 (0.96–1.18)	0.22
		25.98–27.23%	10,752	79.6	1.05 (0.95–1.16)	0.35
		27.24–28.53%	10,712	76.6	1.00	Referent
		28.54–29.55%	10,827	81.1	1.07 (0.97–1.18)	0.21
		29.56–30.78%	10,842	80.8	1.06 (0.96–1.17)	0.23
		30.79–32.21%	10,788	72.6	0.95 (0.86–1.06)	0.35
		32.22–34.20%	10,793	77.4	1.03 (0.93–1.13)	0.63
		≥ 34.21%	10,806	79.2	1.07 (0.97–1.18)	0.18
	Race/Ethnicity	Black	31,661	60.9	1.00	Referent
		White	55,580	90.5	1.54 (1.46–1.63)	< 0.01
		Hispanic	13,388	81.5	1.37 (1.27–1.48)	< 0.01
		Asian	3608	76.8	1.28 (1.12–1.46)	< 0.01
		Am Indian	1784	81.3	1.37 (1.15–1.64)	< 0.01
		Other	428	102.8	1.77 (1.29–2.43)	< 0.01
		Unknown	1457	74.1	1.23 (1.01–1.51)	0.04

## Discussion

The major finding of the study was that women and men in the lowest decile of body fatness had a 20–27% higher relative risk of developing a SF during BCT. Moreover, men in the upper most decile had a 15% greater risk of SFs compared to men in the mean BF decile. The same did not hold for women, however. Gender differences in fat mass and bioavailability of estrogens in young adults [40] may provide a partial explanation for the sex differences at the higher %BF levels. Estrogens have positive effects on bone formation [41] and adipocytes produce estrogens [42]. Thus, women with higher %BF may have similar SF risk to women with less BF because of higher estrogen levels. The increase in SF risk at the lowest BF deciles may be related to frame size. In previous studies, low BF was associated with smaller bone width, reflective of small frames size [43], and individuals with smaller tibia and femur widths were more likely to experience lower body SFs in basic training [16].

To examine the contribution of %BF to the risk of developing a SF during BCT, an equation was derived to predict %BF, as %BF is not routinely measured on recruits. Our hypothesis that there would be an acceptable strength of the association between predicted and actual %BF using BMI, age and sex was confirmed as the resulting equation had an *r* = 0.88 and a standard error of estimate of 4.2%BF, which was very similar to the strength of associations and errors of estimate derived by others [28–33] (see Table 3). Inclusion of race did little to improve the model. Initially it was assumed that race could contribute to the predictability of %BF because of known racial differences in body composition [34]. However, other investigations [29, 31] that have examined or included race/ethnicity in their %BF predictive equations also showed that this variable contributes little to the prediction of %BF when BMI, age and gender were included. The present study did find that individuals self-reporting as black had a lower risk of SFs than other racial groups, a finding that has been replicated in many other investigations [2, 9, 12–15]. The lower SF risk among blacks could be partly related to the higher bone mineral density and thicker bone cortical areas of blacks compared to other racial/ethnic groups [44–47]. The racial difference in bone mineral density between blacks and whites persists after adjustments for body composition, dietary history, sun exposure, biochemical bone markers, lifestyle characteristics, and other factors [47].

The PRESS statistic produced an r-value and SEE that were very similar to that obtained from the original data in the multiple linear regression model. This indicated that that the original model was consistent and that the equation produced an adequate prediction of %BF from BMI, age, and gender in our sample of male and female recruits. Future BCT studies that collect height, weight, age and gender may be able to use this equation to predict %BF.

Although no previous study has examined associations between SF risk and body fat in BCT, three studies have prospectively examined associations between BF and overall BCT injury risk [3, 5, 48]. Jones et al. [3] found a trend for a bimodal relationship between time-loss injuries and skinfold determined %BF in male recruits (*n* = 124) but not female recruits (*n* = 186). Knapik et al. [48] found a trend toward increasing risk of injuries of any type when the lowest DXA-determined %BF tertile was compared with the highest %BF tertile among both men (*n* = 169) and women (*n* = 164). Westphal et al. [5] actually found a trend indicating lower risk of any injury and time-loss injury among groups with the highest and lowest %BF groups compared to women in a mid %BF range (*n* = 156). These equivocal results may be partially explained by the relatively low sample sizes, few injury cases, and likely low statistical power. In the present study, we included 95% of recruits who attended BCT over an 11-year period which included a very large number of SFs (*n* = 17,811).

At least four investigations have examined the association between %BF and SFs in athletes. One study [22] of elite female runners (*n* = 19 reporting SFs and 19 not reporting SFs) found that DXA-determined %BF was lower but not significantly different among women self-reporting a previous history of SFs (16.6% vs 17.4%, *p* > 0.05). Another study [23] of university male and female lacrosse players (*n* = 5 men with and 30 men without SFs; *n* = 7 women with and 42 without SFs) also found no relationship between %BF (determined with a Tanita MC-190 body composition analyzer) and self-reported SFs. A third study of 54 female runners (*n* = 22 with SFs) found no difference in %BF between SF cases (17.0% BF) and non-SF cases (17.7% BF) (*p* > 0.05). Finally, the fourth study [25] matched 19 athletes with diagnosed SFs (by X-ray, bone scan, or magnetic resonance imaging) with 19 healthy athletes without SFs and found no significant difference in DXA-determined %BF between the two groups (*p* = 0.31). Most studies [22–24] relied on self-reports of SFs and all [22–25] had few SF cases, likely making them underpowered to detect a significant relationship between SF and %BF. Further, these studies [22–25] compared average %BF among those with and without SFs and did not compare risk at various %BF levels as reported in the present study.

Several previous studies have examined the ability of BMI, age, gender, and/or race to predict %BF [28–33] and data on these studies and the equations developed are shown in Table 4. Compared to the present investigation, these previous studies used samples that possessed more diversity in terms of age, BMI, and %BF. Yet the equation produced here has similar level of prediction strength as these other studies.

**Table 4:** Equations Estimating %BF from BMI, Age, and Gender

Study	Criterion Method for BF Determination	Participants	Equation for % Body Fat Prediction	r^2^	SEE
Deurenberg et al. [28]	Underwater weighting	749 healthy M & W, 16–83 yr. in Netherlands	−5.4 + 1.20 ● BMI + 0.23 ● age - 10.8 ● sex	0.79	4.1
Gallagher et al. [29]	4 compartment models using dual energy x-ray absorptiometry, doubly-labled water, & underwater weighting	706 M & W, 20–94 yr., BMI < 35 kg/m^2^, residing near NY city	−10.02 + 1.46 ● BMI + 0.12 ● age - 11.61 ● sex	0.67	5.7
Gallagher et al. [30]	4 compartment models using dual energy x-ray absorptiometry, doubly-labled water, & underwater weighting	1626 M & W, 20–97 yr., convenience sample from UK, US, and Japan	64.5–848 ● (1/BMI) + 0.079 ● age-16.4 ● sex + 0.05 ● sex ● age + 39.0 ● sex ● (1/BMI)	0.74	5.0
Jackson et al. [31, 49, 50]	Underwater weighting	679 M & W, 18–61 yr., from 3 US locations	−13.9 + 1.61 ● BMI + 0.13 ● age - 12.1 ● sex	0.75	5.5
Pasco et al. 2012 [32]	Dual-energy x-ray absorptiometry	1766 M & W, 20–74 yr., in Australia	37.8 + 1.62 ● (BMI-mean) -16.7 ● sex - 0.06 ● (BMI-mean)^2^ + 0.02 ● age - 0.17● sex ● (BMI-mean) + 0.03 ● gender ● (BMI-mean) + 0.04 ● sex ● age	0.83	4.1
Gomez-Ambrosi et al. 2012 [33]	Air displacement plethysmography	6123 Caucasians, 18–80 yr. in Spain	−44.988 + (0.503 ● age) + (10.689 ● sex) + (3.172 ● BMI) – (0.026 ● BMI^2^) + (0.181 ● BMI ● sex) – (0.02 ● BMI ● age) – (0.005 ● BMI^2^ ● sex) + (0.00021 ● BMI^2^ ● age)	0.79	4.7
Present Study	Dual-energy x-ray absorptiometry	349 healthy men and women 18–35 yr., in US Army BCT	−7.53 + 1.43 ● BMI + 0.13 ● age - 14.73 ● sex	0.77	4.2

In all models, BMI is in kg/m^2^, age in years, and in all models except Gomez Ambrosi, sex is 0 = women, 1 = men. For Gomez Ambrosi, sex is 0 = men, 1 = female

Abbreviations: *r*^*2*^ explained variance (%), *SEE* standard error of the estimate (% body fat), *US* United States, *UK* United Kingdom, *NY* New York, *BCT* Basic Combat Training, *M* men, *W* women

Limitations to this study should be noted. The %BF body fat equation developed here was based on a sample of Army recruits and the predictive equation is most appropriately applied to this population. Nonetheless, men and women entering the Army come from a wide-ranging cross-section of the US and are broadly representative of individuals of similar age within this national population. The most obvious limitation is that the %BF values in this study were estimated from a predictive equation. Studies should be conducted that measure actual BF in a large group of recruits and follow those recruits through BCT to obtain SF incidence and examine if the relationships established here are confirmed.

## Conclusion

In summary, we developed a useful equation to predict %BF in US Army recruits and showed that this equation (using BMI, age, and sex) was consistent and produced an adequate prediction of %BF. We applied this equation to show that SF risk was higher among male and female recruits with low %BF and among male recruits with higher body fat.

## Abbreviations

%BF: Percent body fat
95%CI: 95% Confidence interval
AFHSB: Armed Forces Health Surveillance Branch
BCT: Basic combat training
BF: Body fat
BMI: Body Mass Index
DMDC: Defense Manpower Data Center
DXA: dual-energy x-ray absorptiometry
ICD-9: International Classification of Diseases
OR: Odds ratio
PRESS: Predictive residual sums of squares
SEE: Standard error of estimate
SF: Stress fracture
US: United States
